# Polypus of Larynx

**Published:** 1852-03

**Authors:** W. Parker


*Polypus of Larynx.* By Prof. W. Parker.—In reviewing this subject we conclude that polypus of the larynx presents the most decided indications for the performance of bronchotomy. The disease is entirely local, and involves no constant local or general condition which can forbid the operation. If resorted to as a palliative measure, it would involve the necessity of the respiration being carried on afterwards through the artificial opening. But to M. Ehrman belongs the honor of having first effected a radical cure by ablation of the offending growth through an external incision ; an operation which the illustrious Stromeyer, President of the Scientific Congress which assembled at Aix la Chapelle, in 1847, declared to be one of the most brilliant achievements in modern surgery. Much of the success in this case is attributed by the operator to the circumstance that the patient was allowed to recover from the depression of the first part of the operation before it was completed. We recommend this operation as indicated by the nature of the case, especially when the polypus has a pedicle, and by the success which has attended its first performance.—*Ibid.*

